# Osteoporosis and Osteoporotic Vertebral Fractures: Breaking the Chain of Osteoporotic Fractures to Increase Healthy Life Expectancy

**DOI:** 10.14789/jmj.JMJ21-0025-R

**Published:** 2021-11-12

**Authors:** REI MOMOMURA

**Affiliations:** 1 Department of Orthopaedic Surgery, Juntendo University Urayasu Hospital, Chiba, Japan; 1 Department of Orthopaedic Surgery, Juntendo University Urayasu Hospital, Chiba, Japan

**Keywords:** osteoporosis, secondary fracture, osteoporotic vertebral fractures

## Abstract

Osteoporosis is an important issue related to life expectancy and healthy life expectancy in Japan, where the super-aging population is growing. Currently, in Japan, some kind of assistance is needed for an average of 10 years at the end of life. In many cases the reason assistance is needed is loss of mobility due to a fracture caused by a fall. When people suffer one fracture due to osteoporosis, they are also more likely to have another fracture, which is called a secondary fracture. Breaking the negative chain of fractures is very important in osteoporosis. In addition, if patients suffer a loss of mobility due to a compression fracture of the spine, this activity cannot be regained even if the fracture is healed. To prevent this from happening, it is also important to heal fractures rapidly, so that patients can quickly return to normal life, thus extending healthy life expectancy.

## Introduction

**Figure 1:**
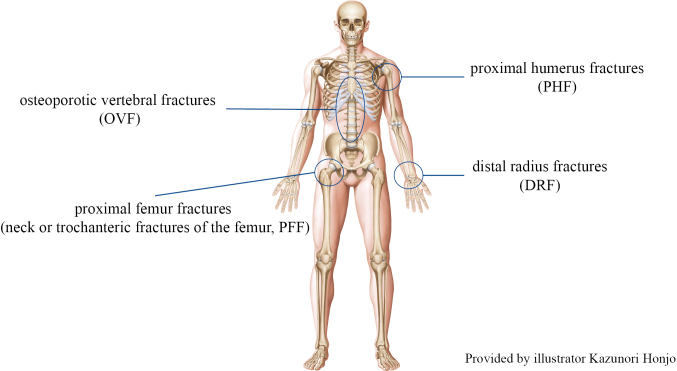
Common sites of osteoporotic fractures

Osteoporosis is an important life-threatening problem in Japan, where there is a growing super-aging population. Patients with osteoporosis are more likely to suffer fractures, such as osteoporotic vertebral fractures (OVF), proximal femur fractures (neck or trochanteric fractures of the femur, PFF), proximal humerus fractures (PHF), and distal radius fractures (DRF) (Figure 1). It has been reported that patients who suffer OVF and PFF in their 60s have a more than ten-fold increased risk of death for up to 2 years^1)^. More specifically, patients who are unable to walk due to a PFF often suffer from various other diseases such as pneumonia, resulting in a 5-year survival rate of 26%. This survival rate is lower than those for liver and stomach cancers. Extending life expectancy is the most important theme in medicine, but we believe that extending healthy life expectancy is just as important. Currently, in Japan, people need some help for an average of 10 years at the end of life, and the reason for needing assistance is more often immobility due to musculoskeletal disorders than weakness due to dementia or old age. In this context, the Japanese Orthopaedic Association (JOA) proposed the term 'locomotive syndrome' to designate a condition requiring nursing care or the risk of developing such a condition, following a decline in mobility resulting from one or more disorders of the locomotive organs, which include the bones, joints, muscles, and nerves^2)^. Even if such support is needed, it is expected that there will be a shortage of manpower in Japan, where one in three people will be 65 or older in 2030. Therefore, we must do all we can to prevent fractures and immobility.

## Secondary fracture

When an osteoporotic fracture occurs, the patient is also more likely to have another fracture. This is called a secondary fracture, and the secondary fracture causes the next fracture, forming a negative chain. For example, in OVF, if a patient suffers from one OVF, the probability of suffering a second OVF is 2.6 times higher, and if there are two OVF, the probability of suffering a third OVF is five times higher^3)^. Similarly, for PFF, the risk of developing a contralateral PFF after one PFF is four times higher than in the general population. Moreover, it is known that one in five people who have a fracture will suffer one on the other side within a year. Guidelines for the prevention of this secondary fracture have now been created. According to these, patients with an OVF or PFF are recommended to start medication for osteoporosis, and when a patient suffers from another osteoporotic fracture, drug treatment is to be initiated if evaluation of bone mineral density (BMD) shows a value of 80% or less of the young adult mean (YAM). It has been reported that the risk can be reduced to less than half by secondary fracture prevention^4)^. Based on this, we investigated the current status of prevention of secondary fractures due to osteoporosis at our institution (Table 1). The subjects were patients who suffered osteoporotic fractures (OVF, PFF, PHF or DRF) between August 2015 and July 2016. We examined whether osteoporosis tests (BMD and/or bone metabolism marker measurement) were performed after the injury and whether osteoporosis treatment was administered. We found that the proportions of patients in whom BMD and/or bone metabolism markers were measured after fracture were: OVF, 58.8%; PFF, 34.0%; PHF, 15.4%; and DRF, 50%. Similarly, the proportions of patients who received osteoporosis treatment after fracture were 75.0%, 36.2%, 38.5%, and 50.0%, after OVF, PFF, PHF and DRF respectively. In Japan, the rate of treatment for secondary fracture prevention after osteoporotic fracture has been reported to be around 15%^5, 6)^, so our results were an improvement. However, we considered that it was necessary to be more proactive in preventing secondary fractures, and thus further strengthen measures against osteoporosis. Consequently more appropriate treatment was commenced according to the guidelines. Then, after the measures were initiated, the same survey was conducted again. The subjects of this survey were patients with the same fractures as above between October 2018 and April 2019. The results showed that the proportions of patients in whom BMD and/or bone metabolism markers were measured after fracture were 54.1%, 11.8%, 14.3%, and 0%, respectively, in the OVF, PFF, PHF and DRF groups, and the proportions given osteoporosis treatment after fracture were 70.3%, 23.5%, 28.6% and 8.3%, respectively. All percentages were below those recorded in the first survey, which made us keenly aware of the difficulty of countermeasures. This depended on the doctor's enthusiasm for treating osteoporosis, so it is necessary to create a system that can be continued regardless of the doctor in charge.

**Table 1:** Percentages of patients undergoing tests for osteoporosis and receiving treatment after osteoporotic fractures

	OVF	PFF	DRF	PHF
Percentage of patients in whom bone density and/or bone metabolism markers were measured after fracture (%)				
2015.8-2016.7	58.8	34.0	15.4	50.0
2018.10-2019.2	54.1	11.8	14.3	0
Percentage of patients who received osteoporosis treatment after fracture (%)				
2015.8-2016.7	75.0	36.2	38.5	50.0
2018.10-2019.2	70.3	23.5	28.6	8.3

There have been other reports of the difficulty of continuing osteoporosis treatment. In a survey of 67,101 patients who received initial administration of osteoporosis drugs in Hokkaido, Japan, from January 2014 to December 2015, it was reported that the retention rate after 1 year was 38.7% and that after 2 years it had fallen to only 7.7%^7)^. At our institution, we surveyed patients who planned to continue weekly osteoporosis treatment for two years in principle, and found that 36.7% of patients quit in just one month, and only 6.7% were able to continue for two years. To understand why patients could not continue with the treatment, we conducted a telephone survey of 39 patients who had stopped taking the medication. The result revealed that elderly people are often unable to attend their appointments due to other diseases. Interestingly, it was also found that 11 patients had discontinued their medication on their own initiative due to concerns about COVID-19. This is a very important issue, because some treatments for osteoporosis can lead to a reduction in BMD and even an increased risk of fractures if left untreated after discontinuation^8, 9)^. Therefore, it is important to continue treatment for osteoporosis. However, the essence of osteoporosis treatment is not to take medicine to increase BMD, but to prevent fractures and prevent immobility due to fractures. Treatment of osteoporosis is not limited to drugs, but there have been studies involving interventions such as reducing the incidence of OVF by strengthening the back muscles and increasing BMD by walking^10, 11)^.

## Osteoporotic vertebral fractures (OVF)

The incidence of OVF has been found to increase rapidly after age 65^12)^. Patients with OVF suffer from two stages of pain. At first, about two months after the injury, the patient complains of sharp pain upon waking, but says it doesn't hurt much if they are sleeping or upright. This is because the fractured part moves when they wake up. Treatment at this stage is to put on a brace to stabilize the fracture and to start medication to promote bone formation. It should be noted that OVF can sometimes be severe, and the collapsed vertebral body can compress the spinal cord and cauda equina nerves, causing paralysis and requiring major surgery. However even if this is not the case and the fracture can be stabilized, the second stage of pain will inevitably follow. Many of the patient's complaints at this time are “I get stiff, hurt, and lean forward when I'm standing or walking for a long time. I want to hold something. It's easier to have a cart for shopping.” This is a low back pain due to muscle fatigue caused by a rounded back due to OVF. This pain is related to the structure of the pelvis and spine. In Japanese patients, the pelvis is tilted forward at approximately 45 degrees. The lumbar spine is lordotic when standing straight in that state. The angle of lordosis is approximately 45 degrees, which is the same as the tilt of the pelvis. When an OVF occurs, the lumbar spine loses its shape and straightens. In response to this, the pelvis tilts forward naturally. This is a condition in which the pelvis compensates for the decrease in lordosis of the lumbar spine. If another OVF occurs in this state, the lordosis of the lumbar spine disappears, and in some cases, the lumbar spine may progress to kyphosis. At this point, the pelvis can no longer compensate and the entire body is tilted forward. In order for people in this state to stand upright, they need to use their back muscle to the fullest. However, this causes the back muscle to become tired after a while. This spinal deformity may be surgically cured, but it is so invasive that it is not practical for most older people. Therefore, the patient must accept this condition. For patients with such conditions, we recommend resting when in pain, sitting when working in the kitchen, and using a walker. This is the state of “locomotive syndrome” mentioned above. Before reaching this state, OVF need to be healed quickly. People have been found to lose 3% of their muscle strength after resting for a day. Decreased activity leads to reduced life expectancy and reduced healthy life expectancy.

**Figure 2:**
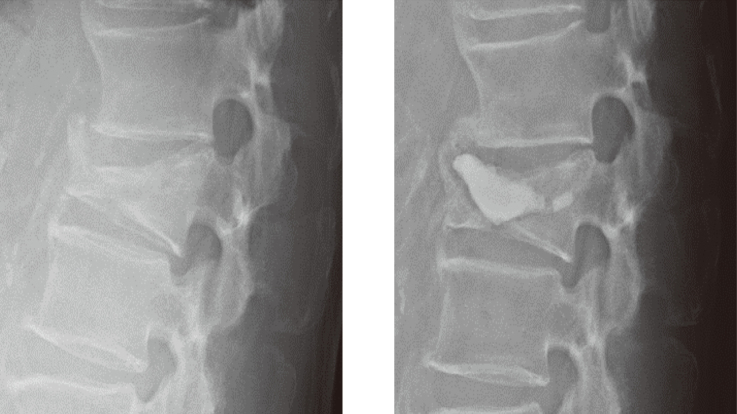
Balloon kyphoplasty for osteoporotic vertebral fracture

What does it mean to heal an OVF quickly? It is important to relieve the pain quickly, for the patient to be able to move from an early stage, and to return to their normal life without losing muscle strength. One of the quickest treatments is an operation called balloon kyphoplasty (BKP) (Figure 2). The crushed bone is inflated with a balloon and cement is injected, allowing the patient to stand and walk the day after surgery. The fracture can be cured very quickly, but in most cases surgery is not immediate due to the amount of preparation required. We have investigated how to cure an OVF quickly. Specifically, we compared the period of improvement in pain caused by OVF between a group treated with BKP and a non-surgery group. The results showed that the average time from the onset of pain to improvement was 12.7 weeks in the surgery group and 10.0 weeks in the non-surgery group, which means that the pain improved faster without surgery. The problem with this is that it takes a long time to perform surgery, and it makes no sense to have surgery early. In fact, other studies have shown that people who have undergone BKP are less likely to develop other illnesses and have a longer lifespan than those who have been cured without surgery^13-15)^. This shows the importance of quickly healing an OVF. Summarizing the treatment of OVF, the initial pain can be expected to improve in about 10 weeks without surgery. During that time, if the patient is unable to move due to pain, the patient's muscle strength and quality of life will deteriorate and cannot be restored. In that case, early surgery should be considered.

In conclusion, it is important to take measures to prevent subsequent fractures from occurring due to osteoporosis, and it is also important to heal a first osteoporotic vertebral body fracture as soon as possible to allow the patient to return to their normal life.

## Funding

No funding was received.

## Author contributions

RM analyzed and interpreted the patient data, and was a major contributor in writing the manuscript.

## Conflicts of interest

The author declare that there are no conflicts of interest.
